# Severe Pediatric Diabetic Ketoacidosis Complicated by Dialysis-Requiring Acute Tubular Injury, in a Child Newly Diagnosed with Type 1 Diabetes Mellitus: A Case Report

**DOI:** 10.3390/reports9030283

**Published:** 2026-08-25

**Authors:** Ali Alamer, Sajjad Alkadhem, Osama Kattih, Ahmed Al-Amoudi, Aida Al Jabri, Maali Alali, Ahmed Soliman

**Affiliations:** 1 Pediatric Intensive Care Unit, Almoosa Specialist Hospital, Al-Ahsa 31982, Saudi Arabia; o.kattih@almoosahospital.com.sa (O.K.); alamoudi-ahmed@hotmail.com (A.A.-A.); dr_aida_s@hotmail.com (A.A.J.); maali1412@gmail.com (M.A.); ahmed.zakzouk@yahoo.com (A.S.); 2 Pediatric Department, Saudi German Hospital, Dammam 32313, Saudi Arabia

**Keywords:** pediatric, DKA, AKI, pancreatitis, microhemorrhages, obesity

## Abstract

**Background and Clinical Significance**: Diabetic ketoacidosis (DKA) is a common presentation of new-onset type 1 diabetes mellitus in children; however, severe DKA complicated by acute pancreatitis, dialysis-requiring acute kidney injury (AKI), severe hypertension, and neurological involvement is uncommon. Early recognition of these complications is essential because they may substantially increase morbidity and complicate standard DKA management; **Case Presentation**: An 11-year-old Saudi girl with morbid obesity (BMI 43 kg/m^2^), previously in good health, was brought to the emergency department after being found semi-conscious. She had experienced intermittent abdominal pain for five weeks and vomiting for four days. On presentation, she was critically ill, dehydrated, and confused (Glasgow Coma Scale 11/15) and exhibited Kussmaul breathing and abdominal tenderness. Laboratory investigations confirmed severe new-onset DKA, with a blood glucose level of 684 mg/dL, pH < 7.0, HbA1c 12.2% and an anion gap > 37 mEq/L. Despite standard DKA management, metabolic acidosis persisted and renal function progressively deteriorated, accompanied by oliguria and severe hypertension reaching 200 mmHg. By day 4, the patient developed anuria and marked creatinine elevation to 560 µmol/L. Brain magnetic resonance imaging demonstrated cerebral microhemorrhages in the setting of multifactorial encephalopathy. Continuous kidney replacement therapy was initiated for KDIGO stage 3 AKI with refractory metabolic acidosis. Autoimmune testing supported the diagnosis of type 1 diabetes mellitus, while renal biopsy demonstrated acute tubular injury; **Conclusions**: This case highlights a rare, severe multisystem presentation of pediatric DKA. Close monitoring for renal, neurological, pancreatic, and hypertensive complications is crucial, particularly when the clinical course does not improve as expected with standard therapy.

## 1. Introduction and Clinical Significance

Despite improvements in pediatric critical care, diabetic ketoacidosis (DKA) is still one of the most dangerous acute consequences of pediatric diabetes mellitus and is still linked to considerable morbidity and mortality. Hyperglycemia, metabolic acidosis, and ketosis are the hallmarks of diabetic ketoacidosis (DKA), according to the International Society for Pediatric and Adolescent Diabetes (ISPAD) [1]. Severe DKA is defined by pH < 7.1 or bicarbonate < 5 mmol/L.

It is increasingly acknowledged that acute kidney injury (AKI) is a serious consequence of juvenile DKA rather than an uncommon side effect. About 40–60% of DKA hospitalizations in large pediatric cohorts have been shown to involve AKI, with increasing severity linked to severe acidosis, corrected hypernatremia, profound dehydration, and hemodynamic compromise [2,3]. Dialysis-requiring AKI is still rare and marks a severe end of the DKA continuum, even though the majority of cases respond with fluid resuscitation and insulin treatment.

Because pancreatic enzymes increase and stomach pain can occur in DKA without actual pancreatic inflammation, the co-occurrence of DKA with acute pancreatitis poses additional diagnostic challenges. According to current standards, a diagnosis of pediatric pancreatitis must meet at least two of three criteria: imaging abnormalities consistent with pancreatitis, pancreatic enzymes larger than three times the upper limit of normal, or distinctive abdominal pain [4].

We report a rare pediatric case of severe autoimmune DKA complicated by acute pancreatitis, acute tubular injury requiring dialysis, severe hypertension, multifactorial encephalopathy with cerebral microhemorrhages, and biopsy results that may indicate renal vulnerability linked to obesity.

## 2. Case Presentation

An 11-year-old previously healthy Saudi girl with morbid obesity was brought to the emergency department after being found semi-conscious at home. Her weight was 90 kg, height 145 cm, and BMI 43 kg/m^2^. The patient had experienced sporadic stomachache for around five weeks prior to four days of continuous vomiting. Fever, respiratory symptoms, drug exposure, intake of herbs, and known infectious contact were all absent.

A sister with obesity and vision impairment of unknown cause, as well as a maternal uncle who had received a renal transplant, made the family history noteworthy. The parents had no medical expenses and were third-degree relatives. She looked extremely sick and terribly dehydrated when she was admitted to the pediatric intensive care unit (PICU). The patient’s vital signs included a temperature of 36.8 °C, heart rate of 113 beats per minute, respiration rate of 24 breaths per minute, blood pressure of 140/90 mmHg, and oxygen saturation of 98% on 5 L per minute. A neurological examination showed disorientation and a Glasgow Coma Scale (GCS) score of 11/15. She exhibited inadequate peripheral perfusion, delayed capillary refill, decreased skin turgor, and dry mucous membranes. Deep Kussmaul breathing was noted. Abdominal examination demonstrated diffuse tenderness radiating to the back and improving on leaning forward.

Initial arterial blood gas showed profound metabolic acidosis with pH < 7.0, non-calculable bicarbonate, and PCO_2_ 19 mmHg. Laboratory evaluation revealed serum glucose 684 mg/dL, positive ketones, HbA1c 12.2%, and an anion gap > 37 mEq/L, confirming severe new-onset DKA. Corrected sodium was 141 mmol/L and serum osmolality 305 mOsm/kg, supporting DKA rather than hyperglycemic hyperosmolar state. Initial creatinine was 115 µmol/L with an estimated Glomerular Filtration Rate (GFR) of 46 mL/min/1.73 m^2^. Serial electrolyte results are shown in Table 1. Further investigations revealed hypertriglyceridemia, leukocytosis, high inflammatory markers, and significantly elevated pancreatic enzymes (lipase 1096 U/L and amylase 429 U/L). Acute pancreatitis was identified based on typical stomach pain and lipase values more than three times the upper limit of normal. A urinalysis revealed microscopic hematuria and significant proteinuria. An ultrasound of the kidneys was normal.

Isotonic fluid resuscitation, deficit replacement, continuous insulin infusion (0.1 units/kg/hour), and electrolyte monitoring with potassium and phosphate replacement were the first steps in the standard care of juvenile DKA. Ketosis and blood glucose levels progressively improved. Severe metabolic acidosis, however, continued despite the biochemical resolution of ketoacidosis. This was accompanied by increasing renal dysfunction and decreasing urine output, indicating growing AKI rather than persistent DKA. Due to prolonged severe acidemia, sodium bicarbonate was given twice, although the relief was only temporary. Additionally, the patient’s hypertension worsened and necessitated hydralazine treatment.

**Figure 1:**
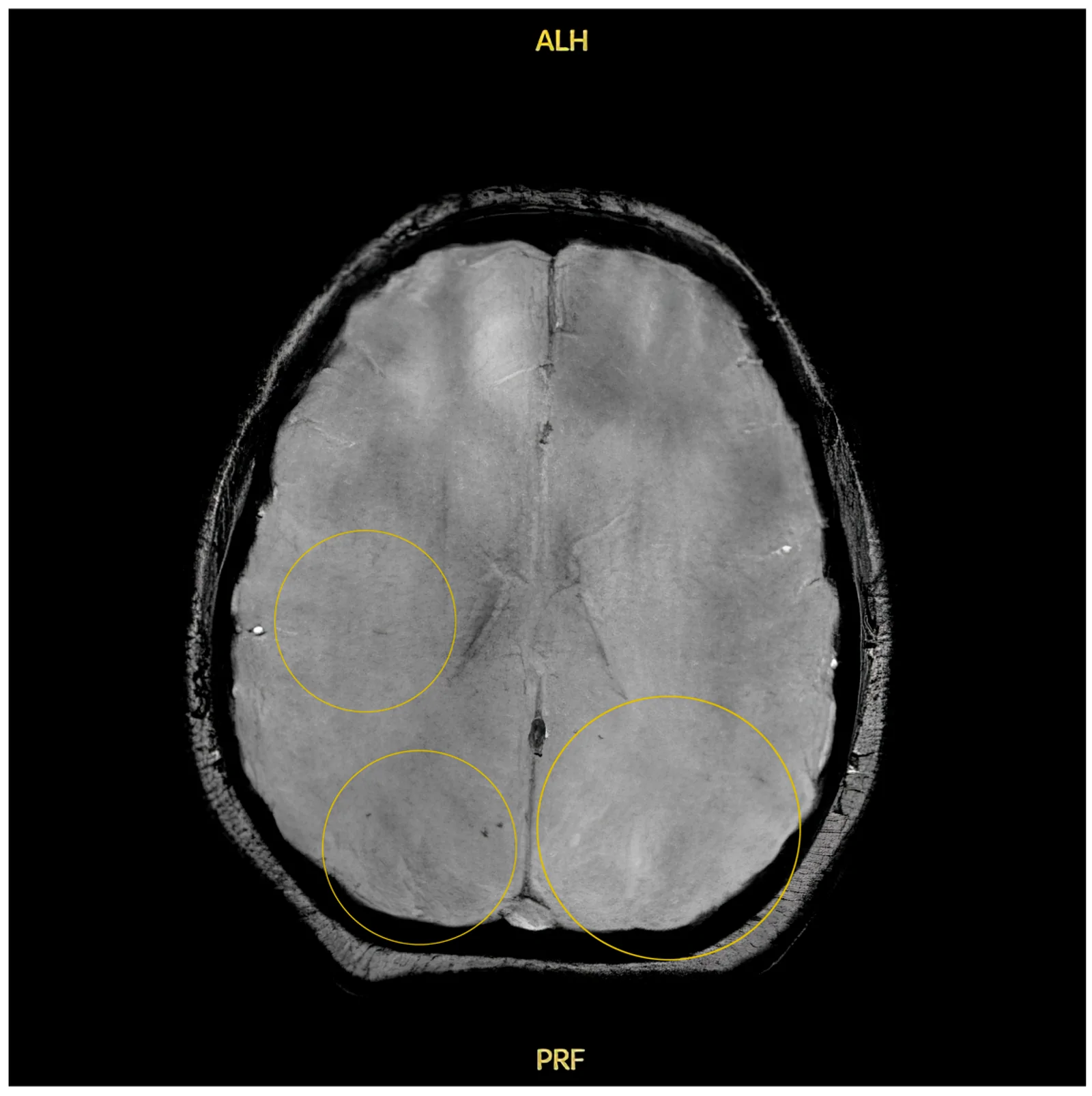
Brain magnetic resonance imaging demonstrates multiple cerebral microhemorrhages involving the bilateral parieto-occipital deep white matter in the setting of severe DKA, uremia, hypertensive crisis, and multifactorial encephalopathy.

By hospital day 3, ketoacidosis had been resolved and insulin was transitioned into a subcutaneous basal-bolus regimen. Despite this, pH remained 7.09 with bicarbonate 7 mmol/L, while serum creatinine increased to 448 µmol/L. Her neurological status fluctuated between GCS 9 and 12/15. On hospital day 4, she progressed to complete anuria with systolic blood pressure reaching 200 mmHg. Serum creatinine rose to 560 µmol/L and urea to 33 mmol/L. Brain MRI demonstrated multiple cerebral microhemorrhages involving the bilateral parieto-occipital deep white matter (Figure 1).

**Figure 2:**
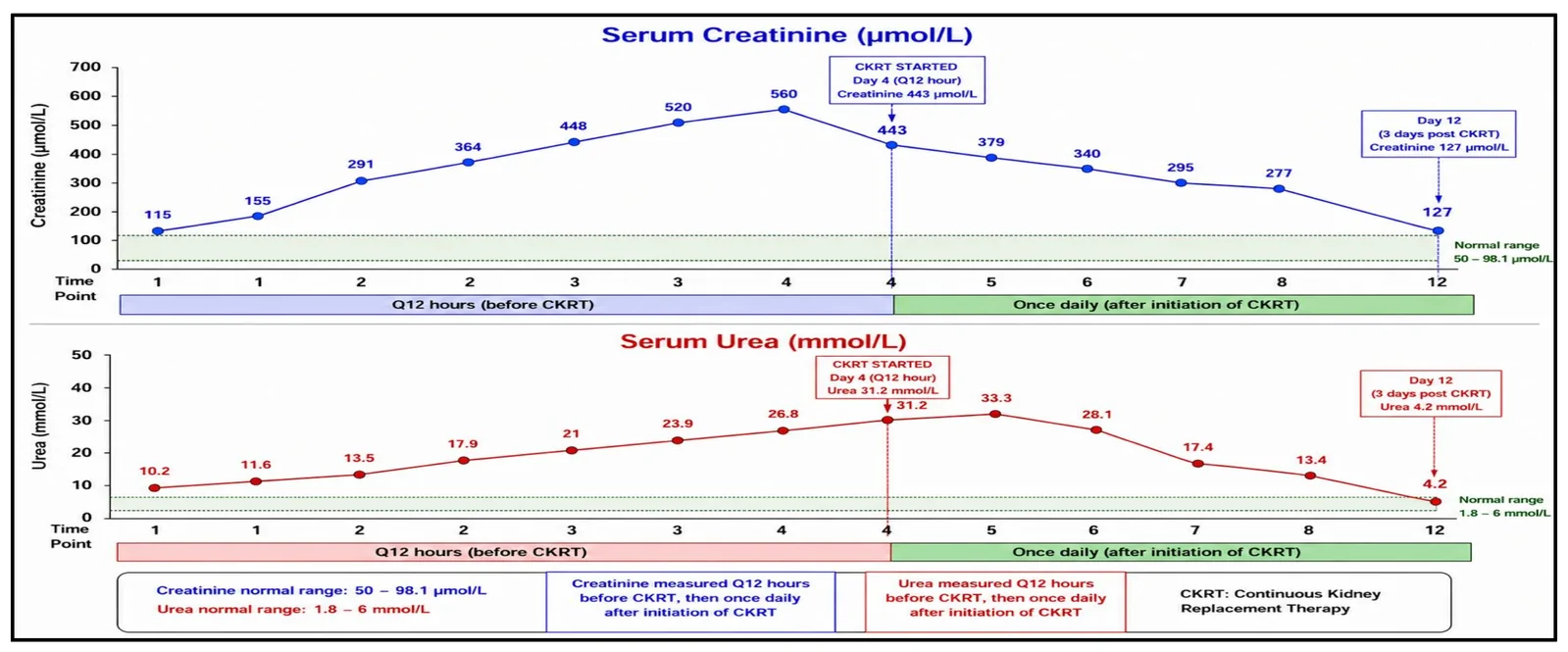
Serial serum creatinine and urea trends showing progressive azotemia before CKRT initiation, followed by biochemical improvement after five days of CKRT and continued renal recovery after discontinuation of extracorporeal support. N.B.: CKRT, continuous kidney replacement therapy; Cr, creatinine; BUN, blood urea nitrogen; µmol/L, micromoles per liter; mmol/L, millimoles per liter.

For Kidney Disease: Improving Global Outcomes (KDIGO) stage 3 anuric AKI, refractory metabolic acidosis, increasing azotemia, severe hypertension, neurological decline, and risk of fluid overload, continuous kidney replacement treatment (CKRT) was started. Using a right internal jugular dialysis catheter and the Prismaflex^®^ (Baxter International Inc., Deerfield, IL, USA) platform with an ST150 high-flux hemofilter, CKRT was administered as continuous veno-venous hemodiafiltration (Figure 2).

Regional citrate anticoagulation was used with calcium monitoring and replacement as required. The prescribed effluent dose was approximately 30–35 mL/kg/hour, and treatment settings were adjusted according to fluid balance, electrolyte status, and acid–base parameters (Table 2).

To rule out renal vascular thrombosis as a cause of severe oligo-anuric AKI, contrast-enhanced abdomen CT was carried out on the second day of CKRT. Without vascular occlusion, arterial or venous thrombosis, obstructive uropathy, or any other acute intra-abdominal pathology, imaging revealed normal renal enlargement.

**Figure 3:**
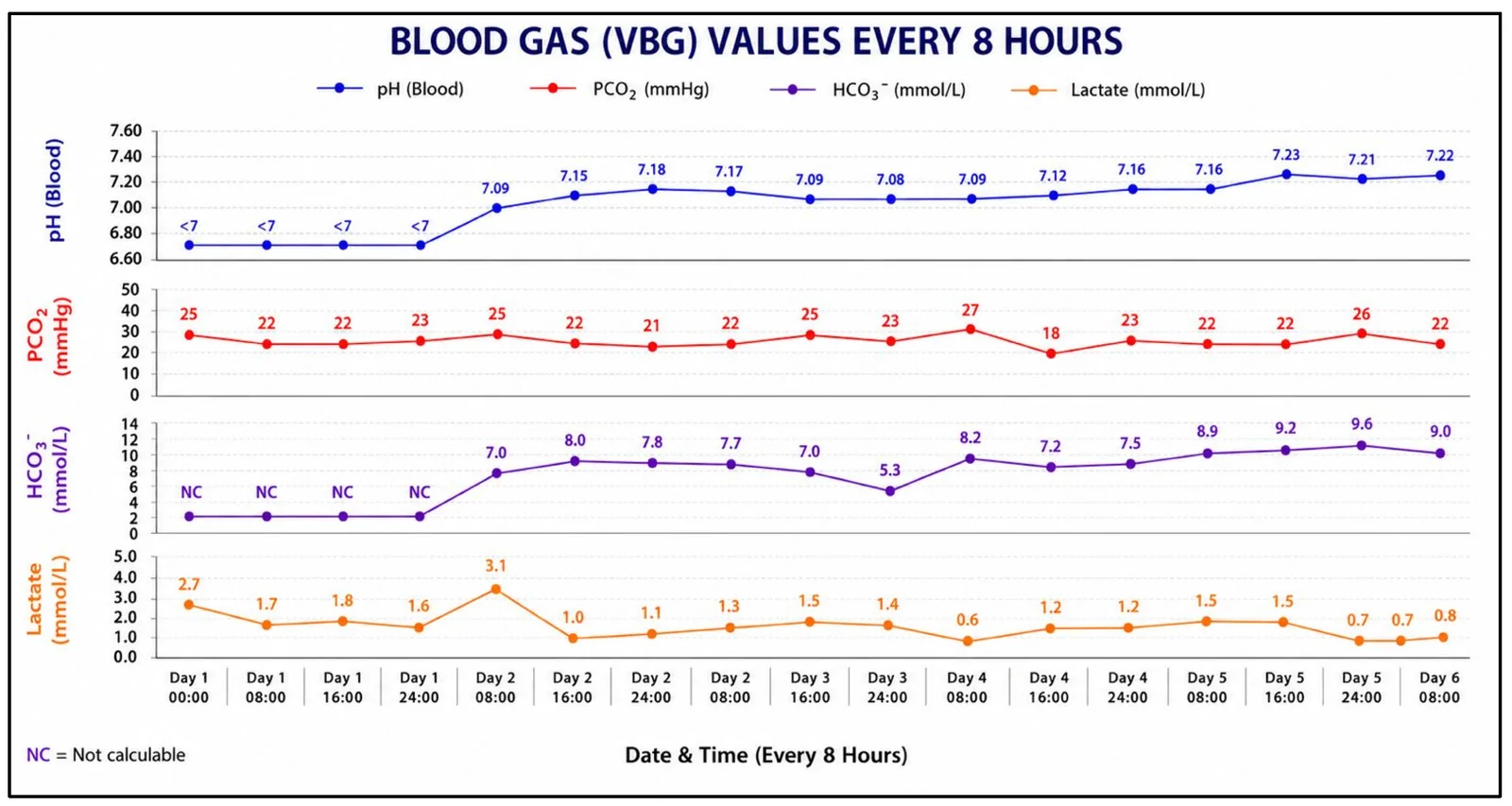
Serial venous blood gas trends before and after CKRT, showing persistent severe metabolic acidosis before extracorporeal support, followed by progressive correction of pH and bicarbonate after CKRT initiation. N.B.: VBG, venous blood gas; CKRT, continuous kidney replacement therapy; pH, potential of hydrogen; PCO_2_, partial pressure of carbon dioxide; HCO_3_^−^, bicarbonate; NC, not calculable; mmol/L, millimoles per liter; mmHg, millimeters of mercury.

Given the severe AKI associated with proteinuria, microscopic hematuria, and hypertension, an extensive autoimmune and glomerular disease workup was performed. ANA, anti-double-stranded DNA, Antineutrophil Cytoplasmic Antibodies (ANCA), anti-Glomerular Basement Membrane (GBM) antibodies, complement levels, rheumatoid factor, anti-Smith antibodies, and anti-endomysial antibodies were negative or normal. Diabetes-related autoimmunity testing demonstrated strongly positive anti-glutamic acid decarboxylase 65 (GAD65) antibodies (670 IU/mL) and mildly positive anti-islet cell antibodies, confirming autoimmune type 1 diabetes mellitus. Metabolic acidosis disappeared, azotemia improved, urine output recovered, blood pressure returned to normal, and neurological state reverted to baseline following five days of CKRT (Figure 2 and Figure 3). The neurological assessment improved to GCS 15/15 between hospital days 5 and 8. By hospital day eight, serum creatinine had dropped to 277 µmol/L, and CKRT was stopped after urine output had recovered and metabolic management was sufficient.

To identify the cause of severe AKI, a percutaneous kidney biopsy was carried out after stabilization. One of the six glomeruli in the specimen had global glomerulosclerosis. Crescents, fibrinoid necrosis, mesangial hypercellularity, thrombotic microangiopathy, and immune-complex deposition were not present. Acute tubular injury (ATI) was indicated by tubular abnormalities such as diffuse interstitial edema, cytoplasmic vacuolation, localized lymphocytic infiltrates, and epithelial simplification. Electron microscopy showed modest mesangial matrix enlargement with focal podocyte foot-process effacement, and immunofluorescence was negative.

**Figure 4:**
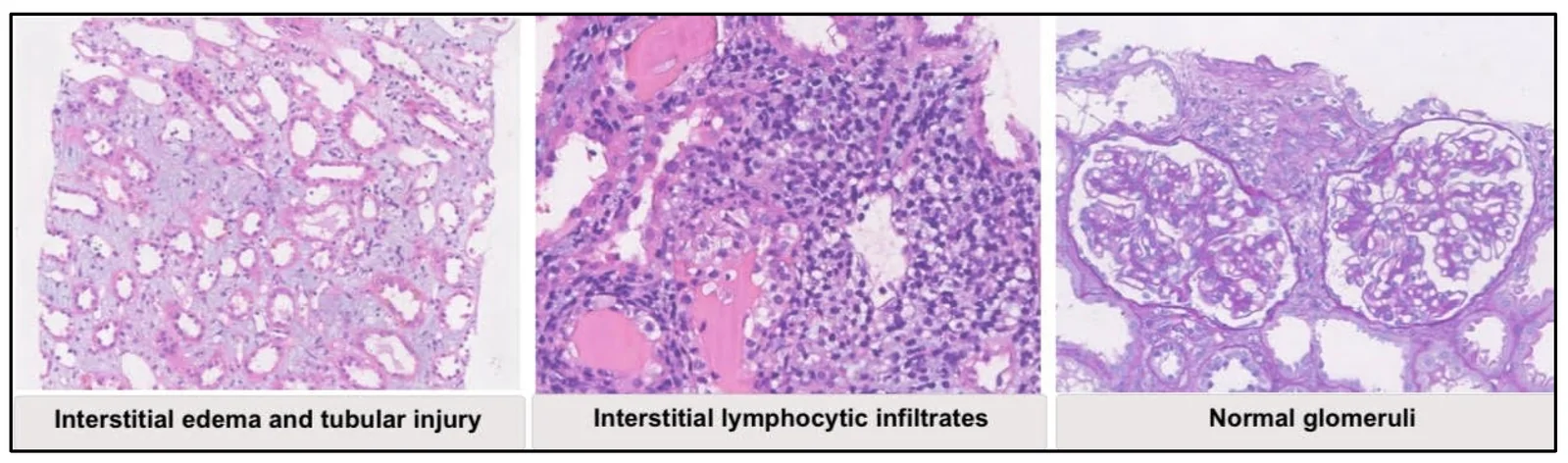
Biopsy confirming acute tubular damage, focal podocyte foot-process effacement, and modest mesangial matrix enlargement. The histopathology images shown in Figure 4 were reproduced from the original diagnostic renal pathology report. The source report did not include scale bar or magnification information.

The biopsies confirmed that the predominant pathological mechanism was acute tubular damage. Proteinuria, focal podocyte foot-process effacement, modest mesangial matrix enlargement, and morbid obesity all coexisted, suggesting potential renal susceptibility linked to obesity. However, the lack of characteristic glomerular lesions and limited tissue collection made it unable to definitively demonstrate obesity-related glomerulopathy (Figure 4).

The patient was transferred from the PICU to the pediatric ward on hospital day 9. At discharge on hospital day 12, serum creatinine had improved to 127 µmol/L, estimated GFR was 42 mL/min/1.73 m^2^, and blood pressure remained normal without antihypertensive therapy. She was discharged home on a basal-bolus insulin regimen consisting of insulin degludec once daily and insulin aspart before meals. At one-month follow-up, renal function had further improved, with serum creatinine at 68 µmol/L and estimated GFR at 81 mL/min/1.73 m^2^.

## 3. Discussion

ATI requiring dialysis, acute pancreatitis, severe hypertension, multifactorial encephalopathy with cerebral microhemorrhages, and potentially obesity-related renal vulnerability complicate this rare and severe presentation of pediatric diabetic ketoacidosis (DKA). The example illustrates a number of crucial clinical and pathophysiological concepts that are pertinent to endocrinology, nephrology, and pediatric critical care.

It is becoming more widely acknowledged that acute renal injury is a serious side effect of juvenile DKA rather than just a temporary side effect of dehydration [2,3]. AKI was seen in about 40–60% of DKA hospitalizations in recent pediatric research, and its severity was closely linked to severe acidosis, intravascular depletion, corrected hypernatremia, hyperosmolarity, and delayed presentation [2,3]. However, progression to KDIGO stage 3 oligo-anuric AKI requiring continuous kidney replacement therapy (CKRT) remains uncommon in children and is generally associated with substantial metabolic and hemodynamic derangement [2,3].

In this case, the primary renal impairment was probably complex. Significant renal hypoperfusion prior to presentation was probably induced by severe osmotic diuresis and extended dehydration. Ischemic tubular damage was probably caused by a combination of systemic inflammation, acute metabolic acidosis, hyperglycemia-induced osmotic losses, persistent vomiting, and poor oral intake. The development from pre-renal hypoperfusion to established intrinsic renal damage is clearly suggested by the passage from modest creatinine increase upon admission to total anuria over a few days.

The distinction between the resolution of ketosis and the continuation of severe metabolic acidosis was a crucial teaching point in this case. Progressive bicarbonate recovery and anion gap closing typically accompany improvement in ketoacidosis after routine DKA therapy [1]. While the patient’s acidity continued to deteriorate with increasing azotemia and oligo-anuria, ketosis and hyperglycemia appropriately improved. This marked a significant diagnostic turning point and indicated that acute renal failure with reduced acid excretion and increasing uremia—rather than ketoacid buildup—was the primary cause of persistent acidemia.

The severe hypertension observed during the course of illness was also clinically significant. Severe AKI may produce progressive sodium and water retention, activation of the renin–angiotensin–aldosterone system, endothelial dysfunction, and impaired autoregulation, all of which may contribute to hypertensive crisis. In this patient, systolic blood pressure reached approximately 200 mmHg during the anuric phase, further increasing concern for hypertensive encephalopathy and secondary neurological injury.

One of the main causes of morbidity and mortality in pediatric DKA is still neurological decline. Although the most well-known neurological consequence is cerebral edema, there is growing evidence that DKA-associated brain injury encompasses a wider range of conditions, including neuroinflammation, endothelial dysfunction, disruption of the blood–brain barrier, impaired cerebral autoregulation, and microvascular injury [1,3]. In the context of acute metabolic disruption and hypertensive crisis, the cerebral microhemorrhages seen on magnetic resonance imaging in this case most likely represent severe microvascular endothelial injury. Cerebral impairment may also have been caused by significant acid–base imbalances and uremia.

The co-occurrence of acute pancreatitis made diagnosis much more challenging. Because pancreatic hypoperfusion and non-specific enzyme leakage can induce abdominal discomfort, nausea, vomiting, and high pancreatic enzymes during uncomplicated DKA, isolated lipase rise is not enough to diagnose pancreatitis [4]. However, in this case, the patient met recognized pediatric diagnostic criteria by exhibiting typical stomach discomfort and a lipase increase that was more than three times the normal upper limit [4]. There was also hypertriglyceridemia. Pancreatic ischemia, oxidative stress, inflammatory cytokine activation, and brief disruptions in lipid metabolism are all possible components of the complex pathogenesis of pancreatitis in DKA [5]. The appearance of microscopic hematuria and nephrotic-range proteinuria early in the clinical course was another noteworthy feature of this case. Rather than isolated pre-renal AKI, these results prompted concerns about intrinsic glomerular disease. Lupus nephritis, vasculitis-associated glomerulonephritis, thrombotic microangiopathy, and rapidly progressing glomerulonephritis were among the differential diagnoses. Nevertheless, thorough autoimmune serologic testing yielded no results, and renal biopsy ultimately ruled out vasculitic renal disease, thrombotic microangiopathy, and immune-complex glomerulonephritis.

Acute tubular damage was shown to be the predominant lesion in the renal biopsy. Ischemic–toxic ATI was supported by histopathological findings such as tubular epithelial simplification, cytoplasmic vacuolation, and diffuse interstitial edema. Crucially, no thrombotic lesions, immune-complex deposition, crescents, or fibrinoid necrosis were found. These results provided compelling evidence against primary inflammatory glomerular disease and in favor of severe tubular injury caused by sustained hypoperfusion and metabolic stress. Additionally, the biopsy revealed potential kidney susceptibility linked to obesity. Morbid obesity is increasingly recognized as an independent risk factor for chronic kidney disease and glomerular hyperfiltration injury in children. Glomerular hyperfiltration, podocyte stress, mesangial enlargement, increasing proteinuria, and ultimately focal segmental glomerulosclerosis are the hallmarks of obesity-related glomerulopathy (ORG) [6]. Although this patient did not demonstrate classic advanced ORG lesions, the presence of morbid obesity, baseline proteinuria, mild mesangial matrix expansion, and focal podocyte foot-process effacement raises the possibility that pre-existing obesity-associated glomerular stress reduced renal reserve and increased susceptibility to severe AKI during DKA.

In this case, the choice to start CKRT was clinically critical. In cases of severe AKI with resistant metabolic abnormalities, progressive fluid overload, severe electrolyte imbalances, or neurological sequelae, early CKRT initiation is increasingly supported by current pediatric critical care practice [7,8]. This patient satisfied several recognized criteria for extracorporeal support, such as severe hypertension, encephalopathy, progressive azotemia, refractory metabolic acidosis, and anuria. While avoiding the quick osmotic changes that can exacerbate neurological damage in severe DKA, CKRT offered regulated acid–base correction and progressive solute clearance.

One significant positive result of CKRT is the patient’s significant renal recovery. However, the patient is more likely to develop chronic kidney disease in the long run if they have obesity, severe AKI, proteinuria, and potential obesity-related renal stress [8]. Since pediatric survivors of severe AKI are increasingly known to have an increased risk for hypertension, chronic proteinuria, hyperfiltration damage, and future development of CKD, longitudinal nephrology follow-up is still crucial [9].

This case emphasizes the importance of maintaining a broad differential diagnosis when the clinical course of DKA deviates from expected metabolic recovery. Persistent severe acidosis after resolution of ketosis, progressive oliguria, refractory hypertension, or neurological deterioration should prompt immediate reassessment for intrinsic renal injury and other systemic complications rather than attributing all abnormalities solely to DKA [10]. Early multidisciplinary involvement, timely renal replacement therapy, and renal biopsy were essential in establishing the final diagnosis and guiding management in this patient.

## 4. Conclusions

In this case, a child with morbid obesity presented with a rare and severe case of new-onset autoimmune pediatric diabetic ketoacidosis complicated by acute pancreatitis, acute tubular damage necessitating dialysis, severe hypertension, and multifactorial encephalopathy with cerebral microhemorrhages. After ketosis is resolved, persistent severe metabolic acidosis should be reevaluated for other reasons, especially intrinsic renal damage. Key signs of severe AKI necessitating early CKRT included progressive oligo-anuria, increasing azotemia, resistant hypertension, and neurological decline. Renal biopsies verified acute tubular damage and ruled out immune-mediated glomerular disease. The results also point to potential renal susceptibility linked to obesity. The diagnosis and effective treatment of pediatric DKA required early multidisciplinary management, underscoring the significance of identifying severe renal and neurological sequelae.

## Figures and Tables

**Table 1:** Serial electrolyte results during hospitalization.

	Na	K	Cl	Mg	tCa	Phosphate
Day 1	137 mmol/L	3.5 mmol/L	109 mmol/L	1.14 mmol/L	2.5 mmol/L	1.12 mmol/L
Day 2	143 mmol/L	3.8 mmol/L	122 mmol/L	0.93 mmol/L	2.53 mmol/L	1.19 mmol/L
Day 3	141 mmol/L	5.1 mmol/L	127 mmol/L	0.8 mmol/L	2.15 mmol/L	1.4 mmol/L
Day 4	144 mmol/L	3.8 mmol/L	128 mmol/L	0.9 mmol/L	2.18 mmol/L	1.26 mmol/L
Day 5	139 mmol/L	3.9 mmol/L	118 mmol/L	0.73 mmol/L	2.12 mmol/L	1.33 mmol/L
Day 7–9	144 mmol/L	4.9 mmol/L	107 mmol/L	0.69 mmol/L	2.1 mmol/L	1.35 mmol/L
Reference Value	135–145 mmol/L	3.5–5.1 mmol/L	98–107 mmol/L	0.66–1.07 mmol/L	2.20–2.70 mmol/L	1.05–1.85 mmol/L

**Table 2:** CKRT prescription and treatment parameters.

CKRT Day	Mode	Blood Flow	Dialysate	Replacement	Net UF
Day 1	CVVHDF	200 mL/min	650 mL/h	650 mL/h	50 mL/h
Day 2	CVVHDF	100–150 mL/min	600 mL/h	250 mL/h	50–70 mL/h
Day 3	CVVHDF	150 mL/min	600 mL/h	600 mL/h	60 mL/h
Day 4	CVVHDF	100 mL/min	600 mL/h	340 mL/h	100 mL/h
Day 5	CVVHDF	50–150 mL/min	700–1500 mL/h	700–1500 mL/h	200–800 mL/h

N.B.: CKRT, continuous kidney replacement therapy; CVVHDF, continuous veno-venous hemodiafiltration; KDIGO, Kidney Disease: Improving Global Outcomes; UF, ultrafiltration; mL/min, milliliters per minute; mL/h, milliliters per hour.

## Data Availability

The original contributions presented in this study are included in the article. Further inquiries can be directed to the corresponding authors upon reasonable request.

## Abbreviations

The following abbreviations are used in this manuscript:

DKA	Diabetic ketoacidosis
AKI	Acute kidney injury
BMI	Body mass index
PICU	Pediatric intensive care unit
GCS	Glasgow Coma Scale
KDIGO	Kidney Disease: Improving Global Outcomes
CKRT	Continuous Kidney Replacement Therapy
Anti-dsDNA	Anti-double-stranded DNA antibodies
ANCA	Antineutrophil Cytoplasmic Antibodies
GBM	Glomerular Basement Membrane
GAD65	Glutamic acid decarboxylase 65
GFR	Glomerular Filtration Rate
ATI	Acute tubular injury
ORG	Obesity-related glomerulopathy
